# miRNAsong: a web-based tool for generation and testing of miRNA sponge constructs *in silico*

**DOI:** 10.1038/srep36625

**Published:** 2016-11-18

**Authors:** Tomas Barta, Lucie Peskova, Ales Hampl

**Affiliations:** 1 International Clinical Research Center, St. Anne’s University Hospital Brno, Pekařská 53, 656 91 Brno, Czech Republic; 2 Department of Histology and Embryology, Faculty of Medicine, Masaryk University, Kamenice 3, 625 00 Brno, Czech Republic

## Abstract

MicroRNA (miRNA) sponges are RNA transcripts containing multiple high-affinity binding sites that associate with and sequester specific miRNAs to prevent them from interacting with their target messenger (m)RNAs. Due to the high specificity of miRNA sponges and strong inhibition of target miRNAs, these molecules have become increasingly applied in miRNA loss-of-function studies. However, improperly designed sponge constructs may sequester off-target miRNAs; thus, it has become increasingly important to develop a tool for miRNA sponge construct design and testing. In this study, we introduce **mi**cro**RNA s**p**on**ge **g**enerator and tester (miRNAsong), a freely available web-based tool for generation and *in silico* testing of miRNA sponges. This tool generates miRNA sponge constructs for specific miRNAs and miRNA families/clusters and tests them for potential binding to miRNAs in selected organisms. Currently, miRNAsong allows for testing of sponge constructs in 219 species covering 35,828 miRNA sequences. Furthermore, we also provide an example, supplemented with experimental data, of how to use this tool. Using miRNAsong, we designed and tested a sponge for miR-145 inhibition, and cloned the sequence into an inducible lentiviral vector. We found that established cell lines expressing miR-145 sponge strongly inhibited miR-145, thus demonstrating the usability of miRNAsong tool for sponge generation. URL: http://www.med.muni.cz/histology/miRNAsong/.

MicroRNAs (miRNAs) are short non-coding RNA molecules that regulate the expression of their target genes at the post-transcriptional level by binding to their respective mRNAs. Upon hybridisation of the miRNA/messenger (m)RNA duplex, the target mRNA is cleaved and degraded or blocked from translation. The miRNAs represent key players in the regulation of multiple genes, and thus, virtually all cell processes, including cell cycle regulation, apoptosis, differentiation, and metabolism^1^,^2^,^3^. Aberrant miRNA expression is also involved in numerous diseases including cancer^4^,^5^, and miRNA-based therapies are under investigation^6^,^7^,^8^,^9^.

The general experimental approach to study the specific role of miRNAs is to perform gain-of-function or loss-of-function experiments. Loss-of-function studies are preferable, as they demonstrate functions that depend on physiological miRNA levels, whereas in gain-of-function studies miRNA over-expression may results in repression of non-physiological target mRNAs^10^,^11^.

Several methods have been described to study the effect of miRNA loss-of-function: antisense oligonucleotide-mediated inhibition, gene knockouts, and miRNA sponges^12^. Antisense oligonucleotides are expensive and not applicable for long-term inhibition due to their degradation and rapid dilution during cell proliferation. Moreover, they are specific to an individual miRNA family member. Thus, it is preferable to use multiple antisense oligonucleotides in order to inhibit an entire miRNA family^10^,^13^,^14^. Generation of miRNA knockouts is challenging, because many miRNAs have seed family members located at multiple different loci in the genome; therefore, these miRNAs should be knocked out individually. Furthermore, numerous miRNAs are transcribed in clusters, which may make it difficult to delete one without affecting other members of the cluster^10^.

MiRNA sponges are exogenously introduced transcripts containing multiple tandem high-affinity binding sites to a miRNA of interest^13^. These miRNA-binding molecules are competitive regulators that sequester specific miRNAs, thus preventing miRNA/mRNA interaction. The sponge sequence consists of multiple miRNA-binding sites (MBS) separated by a 4–6 nucleotide spacer sequence (Fig. 1)^10^,^12^,^13^,^15^. MBSs are either perfectly antisense or contain a bulge at the central position. However, a perfectly base-paired miRNA sponge interaction is vulnerable to interference-type cleavage and rapid sponge degradation, so MBS containing a 4-nucleotide central bulge are more effective^12^,^13^,^15^,^16^,^17^. MiRNA sponges offer the advantage of inhibiting all seed family members and, when multiple MBSs are introduced, miRNA sponges can be used to inhibit a whole miRNA cluster^15^. Given their advantages over other means of miRNA inhibition, miRNA sponges have the potential for use in disease treatment^18^,^19^,^20^.

It should be noted that miRNA sponges may exhibit off-target effects by sequestering unspecific miRNAs. As the human genome contains thousands of miRNA sequences^21^ and given the small size of each mature miRNA (∼22 nucleotides), there is a high probability that poorly designed miRNA sponge constructs may exhibit unspecific sequestration of miRNAs. Therefore, sponge sequences must be carefully optimised to avoid the binding of other miRNAs.

At present, there is a lack of software tools for miRNA sponge construct design and for off-target testing. In order to address this issue, we developed a web-based tool, **mi**cro**RNA s**p**on**ge **g**enerator and tester (miRNAsong), which is freely available at: http://www.med.muni.cz/histology/miRNAsong. This tool allows the user to generate miRNA sponge sequences specific to a target miRNA, miRNA family and/or cluster. It also has the ability to test sponge sequences *in silico* for potential off-targets in 219 species covering 35,828 mature miRNA sequences (at the time of manuscript submission). Furthermore, we experimentally verified our tool; using miRNAsong, we generated and optimised a miRNA sponge construct for inhibition of miR-145. We cloned the generated sequence into an inducible lentiviral vector and established HEK293T cell lines that expressed the miR-145 sponge upon induction using the small molecule, 4-Isopropylbenzoic acid (cumate)^22^,^23^.

## Results

The miRNAsong tool is a user-friendly, freely available web-based tool for generation and testing of miRNA sponge constructs. It allows the user to: **I)** generate and test miRNA sponges for known, specific miRNAs, **II)** generate and test miRNA sponges using user-defined miRNA sequences, **III)** generate and test miRNA sponges for multiple miRNAs, such as families and clusters, and **IV)** test user-defined miRNA sponge sequences.

### miRNA sponge sequence generation

The miRNAsong tool allows the user to search for specific miRNAs using either the miRNA name or accession number. The list of miRNAs has been downloaded from mirbase.org^24^,^25^,^26^,^27^ and modified to include miRNA names, accession numbers, mature sequences, and species. In addition to searching for specific miRNA sequences, miRNAsong allows for generation of a sponge construct from a miRNA sequence defined by the user.

The miRNAsong tool allows the user to apply various settings when designing a sponge sequence; the user is able to create a perfect antisense MBS or a bulge on the MBS. When the bulge option is selected, a bulge is created at a user-defined site of the MBS. The program generates perfect miRNA antisense sequences, and using an algorithm of nearest-neighbour energy model^28^, it creates a 4-nucleotide mismatch on the target site in such a way that the chances of pairing are minimal, including G-U wobbling^12^,^15^. One nucleotide on the sponge sense strand is then deleted to generate a bulge. The user may also define the number of MBSs in the sponge construct and set the spacer sequence between individual MBSs.

### miRNA sponge sequence testing

The sponge sequence is tested for interaction with miRNAs from a selected organism using RNAhybrid code, developed by Rehmsmeier *et al*.^29^. We chose this algorithm for several reasons: it is fast enough to search large databases of potential target sequences^29^,^30^, it allows for visualisation of interaction sites, it provides minimal free energy of the miRNA/sponge interaction, and it allows for the application of user-defined cut-off criteria. RNAhybrid was originally developed to predict multiple potential binding sites of miRNAs in large target RNAs^29^,^30^. We modified the output of RNAhybrid to display the whole miRNA sponge construct, including all interactions with miRNAs, and compiled the source code on the server-side. The miRNAsong program tests every miRNA sequence from a selected organism for interaction with the sponge sequence. For prediction of optimal miRNA/sponge interaction, the user may define seed region features: canonical (6-mer seed (2–7 nucleotides), 7-mer seed (2–8 nucleotides), offset 6-mer seed (3–8 nucleotides)) or non-canonical miRNA/sponge interaction. When seed region feature option is selected, it forces miRNAsong to show interacting miRNAs that form perfect match with the sponge in a selected seed region. Based on user-defined cut-off settings and seed region features, miRNAsong then displays each miRNA/sponge interaction, including the calculated free energy. Each interacting miRNA with the sponge is then scored by the sum of the free binding energy of all interactions with the sponge. In addition to a detailed graphical output displaying miRNA/sponge interactions, miRNAsong also generates final sponge sense and antisense sequences for easy oligonucleotide ordering.

If the generated sponge sequence does bind off-target miRNAs with similar free binding energy, variations in the sponge construct sequence should be made. MiRNAsong provides a detailed graphical output displaying all sites of the miRNA/sponge interactions. Based on these off-target interaction sites, the user may introduce specific modifications to the sponge sequence, including changing the nucleotide composition of the: **I)** spacer sequence, if the off-target interacts at the spacer site; **II)** bulge sequence or position of the bulge, and **III)** linker sequence.

### Generation of a sponge sequence to inhibit a whole miRNA family or cluster

In order to generate a sponge construct for inhibition of multiple miRNAs (e.g. miRNA families or clusters), miRNAsong allows the user to insert multiple miRNA sequences, including spacer sequences, between individual MBSs, then the program generates and tests the sponge sequence as described above.

### An example of miRNAsong usage and experimental data

In order to provide detailed information on how to use the miRNAsong tool, including construction and cloning of the sponge sequence into the vector, we generated and *in silico* tested a sponge for miR-145 using miRNAsong and cloned the sponge sequence into an inducible vector.

We generated and tested a miRNA sponge sequence for hsa-miR-145-5p using miRNAsong. The sponge sequence included two MBSs, a bulge at nucleotide position 10–13, and a spacer sequence, AATT, between individual MBSs. In silico test for off-targets revealed that the generated sequence binds only miR-145-5p (free energy −69.5 kcal/mol) (settings: −25 kcal/mol cut-off and canonical 6-mer seed). We cloned the generated sponge sequence into an inducible vector as described in the Material and Methods. Sanger sequencing revealed that the sponge sequence included 4xMBS in the correct order (Supplementary Data 1).

**Figure 2:**
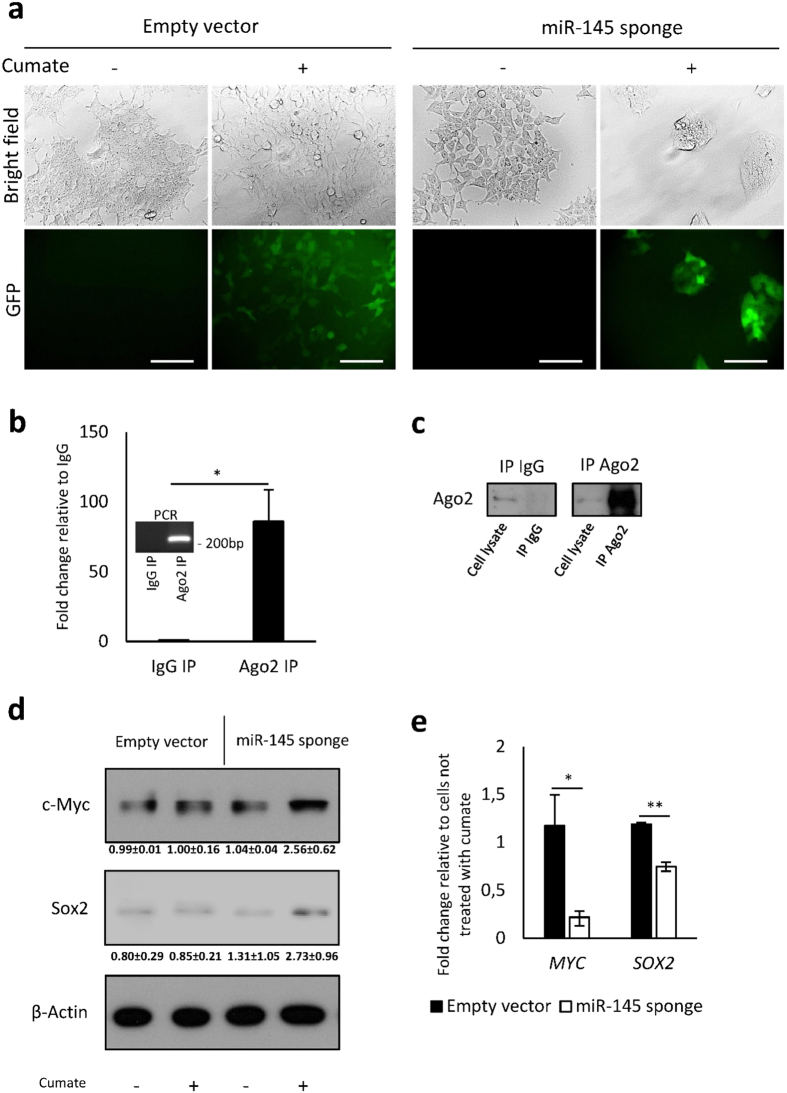
Expression of miR-145 sponge upon cumate induction and efficiency of miR-145 inhibition. (**a**) Cell morphology and GFP expression upon cumate-induced expression of miR-145 sponge, as observed by light and fluorescent microscopy. Scale bars represent 100 μm. (**b**) Quantification of sponge transcript levels in Ago2-immunoprecipitated (Ago2 IP) and control IgG (IgG IP) fractions, as demonstrated by RT-qPCR. Data are depicted as mean + SD of triplicates. The length of the transcript was checked by standard agarose electrophoresis (graph inset). Full-length gel is presented in Supplementary Fig. 1. (**c**) Western blot analysis of Ago2 levels in Ago2 IP and control IgG IP fractions. Full-length blot is presented in Supplementary Fig. 2. (**d**) Expression of c-Myc and Sox2 upon miR-145 sponge induction, as determined by western blot analysis. Experiments were performed on three different HEK293T clones expressing miR-145 sponge or empty vector. One representative western blot is shown. Full-length blot is presented in Supplementary Fig. 3. Quantification of western blots is provided under each western blot. Data represents fold change relative to an empty vector control without cumate ± SD. β-Actin was used as a loading control. (**e**) Levels of *MYC* and *SOX2* transcripts associated with Ago2 upon miR-145 sponge expression. Data are depicted as mean ± SD of triplicates.

The generated lentiviral particles containing the sponge construct or the empty vector were used to infect HEK293T cells. We induced the expression of the sponge using 50 μg/ml of cumate and observed the cells for green fluorescent protein (GFP) expression using fluorescence microscopy. GFP positive cell clusters were manually isolated and expanded. For further analysis, cells were cultured in the presence of cumate for at least 5 days. Cells expressing empty vector and cells cultured in the absence of cumate were used as a control. We observed that upon induced miR-145 sponge expression cells changed their morphology and formed compact clusters, when compared to cells expressing empty vector and/or cells cultured in the absence of cumate (Fig. 2a).

To prove the functionality of miR-145 sponge, we have first determined if the miRNA-containing RNA-induced silencing complex (RISC) was associated with the miRNA sponge transcripts. We tested if the sponge transcripts were enriched in immunoprecipitated (IP) argonaute (Ago)2, a RISC catalytic component. RT-qPCR revealed that the sponge transcripts were strongly enriched in the Ago2-IP fraction compared to the IgG control (Fig. 2b,c and Supplementary Figs S1 and S2), indicating that the sponge transcripts were associated with Ago2-containing RISC complex. As an additional control, we used cells expressing empty vector, however we could not detect the sponge transcript in either Ago2-IP or IgG fractions (data not shown).

Next, we analysed the effect of miR-145 sponge expression on miR-145 target genes. Many genes are regulated by miR-145-5p, including *SOX2* and *MYC*, which belong to the most often studied miR-145-5p targets^1^,^31^,^32^,^33^,^34^. Western blot analysis of Sox2 and c-Myc revealed that both miR-145 targets are up-regulated upon induction of the sponge (Fig. 2d and Supplementary Fig. S3). To further prove the effectiveness of the sponge, we assessed the levels of *MYC* and *SOX2* transcripts in Ago2-IP fraction. RT-qPCR revealed that both transcripts were less enriched (*MYC* ~22% and *SOX2* ~74%) in Ago2-IP fraction upon sponge expression (Fig. 2e) indicating that more *MYC* and *SOX2* transcripts are free to be translated into protein.

Taken together our results indicate that sponge for miR-145 inhibition designed by miRNAsong binds to Ago2-containing RISC complex (similar to endogenous miRNA targets), up-regulates miR-145 target genes *MYC* and *SOX2*, as demonstrated by elevated protein levels of c-Myc and Sox2, and decreased levels of *MYC* and *SOX2* transcripts associated with RISC complex.

## Discussion

An increasing number of studies using sponges for miRNA inhibition have shown that this technique is promising for *in vitro* and *in vivo* experiments, as well as for clinical applications^19^,^20^,^35^. However, improperly designed sponge sequences may sequester off-target miRNAs, leading to possible false-positive results and/or off-target effects. To date, there is a lack of software tools for sponge design and *in silico* testing.

Here, we have presented a web-based tool for *in silico* design and testing of miRNA sponge constructs. There are other available tools that can be used for sponge testing, but they have several disadvantages. The PITA tool, developed by Eran Segal’s group^36^, was originally developed to scan UTRs for possible miRNA targets and can potentially be used for sponge testing. However, this program appears to be outdated (last update was in 2008), allowing it to scan in only 4 species and for only 470 human miRNAs, whereas miRNAsong scans in 219 species and for 2,588 human sequences. Further, the PITA tool is limited to 100 targets per run, as it uses a more complex, slower-folding algorithm, RNAfold, from the Vienna RNA package^37^. It also does not generate graphical output displaying miRNA/mRNA interaction sites; therefore, it may be difficult to introduce specific changes to the sponge sequence in order to decrease off-target binding. STarMiR^38^, developed for the prediction of miRNA-binding sites on RNA from crosslinking immunoprecipitation studies, can also be used. However, STarMiR requires the user to enter each miRNA individually, thus making sponge testing very difficult. Another means of sponge testing, miRSponge, which is a manually curated database for experimentally supported miRNA sponges, was recently developed^39^. This tool has the advantage of being able to search for experimentally validated miRNA sponge sequences, so it does not allow searching for off-targets and does not provide any information on novel or not-yet experimentally verified miRNA sponges. Taken together, in contrast to miRNAsong, the above mentioned tools do not generate miRNA sponge sequences and present considerable disadvantages for sponge construct testing. Our tool allows the user to search for miRNA/sponge interactions in a wide range of species, including all currently known miRNA sequences (based on sequences published on mirbase.org), and provides detailed graphical output of miRNA/sponge interaction sites, thus allowing a user to introduce changes into the sponge sequence when off-target binding occurs. Finally, miRNAsong is fast (for runtime comparison with RNAfold algorithm see ref. 29) and allows for an unlimited number of targets per run.

One could note that the algorithm used in RNAhybrid predicts also non-canonical (out of seed region) interactions between miRNA and sponge, and thus it may predict interactions that are very unlikely to happen. This prevailing presumption of necessity of perfect Watson-Crick pairing in the seed region (2–8 nucleotides at miRNA) has been questioned by many studies (for references see review ref. 40). Recent studies using transcriptome-wide methods of mapping miRNA binding sites revealed that a large proportion of miRNA/target interactions are mediated also through non-canonical sites, thus representing a limitation of ability to delineate a general principle for miRNA/target interactions. For example, in plants a near-perfect base pairing between miRNA/target is crucial, while in animals is rare^41^. To address this problem, miRNAsong allows the user to choose the features of a seed region including disallowance of any seed region at miRNA molecule to reveal non-canonical binding.

Using miRNAsong, we generated and *in silico* tested sponge sequences for miR-145 inhibition. We also cloned the construct into an inducible vector and generated stable cell lines expressing miR-145 sponge. RT-qPCR and western blot analysis showed that miR-145 sponge transcript associates with Ago2 and inhibits miR-145. MiR-145 has been recently linked with stemness and cancer. It has been demonstrated that inhibition of miR-145 has led to elevated expression of stem cell markers in both somatic and differentiated cells^1^,^42^, whereas up-regulation of miR-145 has led to down-regulation of stem cell markers in cancer cells, human embryonic stem cells, and neural stem cells^33^,^42^,^43^,^44^. We have recently shown that miR-145 inhibition in human neonatal fibroblasts leads to elevated expression of Sox2, c-Myc, Klf4, and it facilitates reprogramming to induced pluripotent stem cells^1^. Furthermore, miR-145 inhibition led to a change of cell morphology of fibroblasts towards epithelial shape. In this study, we demonstrate that miR-145 inhibition leads to elevated expression of c-Myc and Sox2 in HEK293T cells, thus corroborating previously published results. We also tested the expression of other stem cell markers (Oct4 and Klf4), but we did not observe any changes in their expression upon miR-145 inhibition (data not shown) presumably due to I) different expression of miR-145 in both cell types, II) different expression of miR-145 targets, and III) distinct nature of HEK293T cells, when compared to fibroblasts. Furthermore, we also observed prominent changes of cell morphology of HEK293T cells upon miR-145 inhibition. This formation of compact clusters of cells with epithelial morphology may be attributed to the elevated expression of Sox2 and c-Myc^1^.

It should be noted that the number of MBSs may strongly influence miRNA inhibition. Kluiver *et al*. reported that 6 MBSs appeared to maximally inhibit miR-19 in WEHI-231 cells; however more MBSs do not necessarily translate into more effective inhibition, because 12 MBSs in a sponge construct were found to be less effective^15^. In addition, other studies have demonstrated that the number of MBSs (typically ranging from 4 to 16 nucleotides) does influence target miRNA inhibition^10^,^45^. In this study, we did not test the correlation between miRNA inhibition effectiveness and numbers of MBSs in the sponge construct. Still, we show that 4 MBSs were sufficient to strongly inhibit miR-145 in HEK239T cells and we do not exclude the possibility that a higher number of MBSs may lead to more effective miRNA inhibition. However, there are several factors that may affect the correlation between the number of MBSs and inhibition effectivity and may also explain discrepancies in effective numbers of MBSs among published studies. For example, promoters used for sponge expression may largely influence the level of sponge transcripts and thus the effectivity of target miRNA inhibition. Sponge transcripts containing multiple MBSs may undergo degradation, and the expression level of the target miRNA in different cell lines can also affect the effectivity of miRNA inhibition. Therefore, there is no general rule regarding how many MBSs should be in a sponge construct, and it appears to be dependent on many aspects, including the promoter used, the cell line, and the expression of the target miRNA.

In should be noted that other alternative approaches are currently used to efectively inhibit miRNAs: I) TuDs (“tough decoys”), similarly to miRNA sponges, contain also MBSs, but they are placed in a single-stranded region of short stem-loop^17^,^46^; II) antagomiRs - contain sequence with full complementarity to the target miRNA(s)^47^; III) “mask” RNA - that binds to the target mRNA and protects it from recognition by the miRNA^48^. Bak and colleagues performed a side-by-side comparison of seven different DNA-encoded miRNA inhibitors including antagomiRs, TuDs, miRNA sponges, and “mask” RNA and concluded that TuDs and bulged miRNA sponges are the most potent miRNA inhibitors^49^.

The miRNAsong program represents a powerful tool for miRNA sponge design and *in silico* testing. We plan to regularly update the list of species and mature miRNA sequences according to new updates on mirbase.org.

## Materials and Methods

### Cell culture

HEK293T cells were cultured in Knockout Dulbecco’s modified Eagle’s medium (DMEM) (Invitrogen, Life Technologies Ltd.) containing 10% foetal bovine serum (PAA), 2 mM L-glutamine (Invitrogen, Life Technologies Ltd.), 1× MEM non-essential amino acid solution, 1× penicillin/streptomycin (PAA), and β-mercaptoethanol (BME) (Sigma-Aldrich).

### miRNA sponge design and cloning

**Figure 3:**
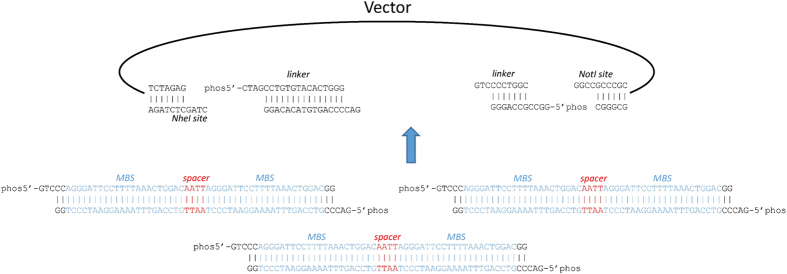
Strategy to ligate miR-145 sponge oligo duplexes into the vector. MBS, miRNA-binding sites.

In order to enable sponge directional cloning, we added 5′-GTCCC to the sense and 5′-GACCC to the antisense strand of the miR-145 sponge sequence generated by miRNAsong (Fig. 3). The resulting oligonucleotides: phos5′-GTCCCAGGGATTCCTTTTAAACTGGACAATTAGGGATTCCTTTTAAACTGGACGG for the sense strand and phos5′-GACCCGTCCAGTTTAAAAGGAATCCCTAATTGTCCAGTTTAAAAGGAATCCCTGG for the antisense strand were ordered as oligonucleotides (Sigma-Aldrich). Oligonucleotides were hybridised by dissolving to 100 μM in 10 mM Tris-Cl (pH 8.5), mixed at a 1:1 molar ratio, and boiled for 10 minutes in a water bath, then allowed to cool for 30 minutes at room temperature.

### Vector preparation

The pCDH-CuO-MCS-IRES-GFP-EF1-CymR-T2A-Puro All-in-one inducible internal ribosome entry site (IRES) vector (System Biosciences, Inc.) was digested using NheI and NotI enzymes, then dephosphorylated using Antarctic Phosphatase (all enzymes from New England BioLabs, Inc.). Linkers (Fig. 3) were hybridised as described above. The whole constructs (vector + linkers + sponges) were ligated in one reaction using a vector:linker ratio of 1:3 and a vector:sponge ratio of 1:1000. The ligation reaction was performed using T4 DNA ligase (New England BioLabs, Inc.). After ligation of multiple inserts and vector, the ligation mixture was used as a template for PCR using primers flanking the cloning site of the plasmid (forward primer: 5′-CCGATCTGGCCATACACTTGA, reverse primer: 5′-AGACCCCTAGGAATGCTCGT)^50^. The fragment containing four MBSs was then gel purified, digested with NheI and NotI, and inserted into the digested vector as described above.

### Lentiviral production and infection

HEK293T cells were transfected with the vector containing the miR-145 sponge or empty vector, as well as plasmids for lentivirus production, pMD2.G (Addgene plasmid #12259), pRSV-Rev (Addgene plasmid #12253), and pMDLg/pRRE (Addgene plasmid #12251) (all were a gift from Didier Trono), and incubated at 37 °C. Culture medium was harvested and changed every 12 hours for a total of 48 hours. Virus supernatant was centrifuged at 4,500 × *g* for 10 minutes and filtered through a 0.45-μm low protein-binding filter. The medium containing the lentiviral particles was mixed with Polybrene (Sigma-Aldrich) at a final concentration of 5 μg/ml and applied to HEK293T cells overnight. The next day, the culture medium was supplemented with 1 μg/ml of puromycin for cell selection. In order to manually select positive clones, we induced sponge expression by adding cumate (final concentration of 50 μg/ml) (Sigma-Aldrich) prepared as 2,000× stocks (100 mg/ml) in 96% ethanol. After 4–5 days, GFP-positive clones were manually selected and propagated in the absence of cumate.

### Induction of sponge expression

In order to switch on expression of the miR-145 sponge, cells were cultured in the presence of cumate at a concentration of 50 μg/ml for at least 5 days. Cells were then examined for GFP expression using fluorescent microscopy and harvested for downstream analyses. As a control, cells cultured in the absence of cumate and cells expressing the empty vector were used.

### Ago2 IP and PCR

Cells were washed three times with phosphate-buffered saline (PBS) (pH 7.4) and UVC-irradiated (0.3 mW/cm^2^ for 45 seconds). Cells were then lysed in buffer containing 50 mM Tris-HCl (pH 7.4), 150 mM NaCl, 5 mM MgCl_2_, 0.5% NP-40, 15 mM ethylenediaminetetraacetic acid (EDTA), freshly added complete mini EDTA-free protease inhibitor cocktail (Roche), and RNAse inhibitor (final concentration 2 U/ml) (Applied Biosystems). Cell lysates were cleared by centrifugation at 15,000 × *g* for 10 minutes at 4 °C, the supernatant was pre-cleared by incubation with protein G sepharose beads (GE Healthcare) on a rotating platform for 1 hour at 4 °C, and the sepharose beads were centrifuged. The supernatant was then incubated on ice for 1 hour with primary antibody EIF2C2 (H00027161-M01, Abnova); normal mouse IgG (sc-2025, Santa Cruz Biotechnology) was used for a control IP reaction. Immunocomplexes were collected overnight at 4 °C on protein G sepharose beads and washed five times with lysis buffer. In order to detect Ago2, immunocomplexes were subjected to western blot analysis using Ago2 primary antibody (#2897, Cell Signalling Technology). RT-qPCR was performed with RNA isolated with Trizol reagent and reverse transcribed using First Strand cDNA Synthesis Kit (Roche) with random hexamer primers. RT product was amplified by real-time PCR (Roche LightCycler^®^ 480 PCR instrument) using LightCycler^®^ 480 SYBR Green I Master kit. The following set of primers was used for detection of sponge expression: forward 5′-TGTACACTGGGGTCCCAGGGATT, reverse 5′-GCACACCGGCCTTATTCCAA. The forward primer annealed to the left linker and 12 nucleotides of the sponge and the reverse primer annealed downstream of the cloning site, thus resulting in specific detection of the miR-145 sponge transcript. The sponge enrichment in IP Ago2 was normalised to the IgG control using the formula −2^−(CtAgo2 − CtIgG)^.

For detection of *MYC* and *SOX2* transcripts associated with Ago2, 1 μg of RNA from Ago2 IP reaction was reverse transcribed using First Strand cDNA Synthesis Kit (Roche) with anchored-oligo(dT) primers. RT product was amplified by real-time PCR (Roche LightCycler^®^ 480 PCR instrument) using LightCycler^®^ 480 SYBR Green I Master kit. Primers were used as follows: *MYC* Left: GCTGCTTAGACGCTGGATTT, *MYC* Right: TAACGTTGAGGGGCATCG; *SOX2* Left: GGGGGAATGGACCTTGTATAG, *SOX2* Right: GCAAAGCTCCTACCGTACCA.

### Western blot analysis

Cells were washed three times with PBS (pH 7.4) and lysed in lysis buffer containing 1% sodium dodecyl sulfate (SDS), 100 mM Tris-HCl (pH 6.8), and 20% glycerol. Protein concentrations were determined using the DC Protein Assay Kit (Bio-Rad) and lysates were supplemented with bromophenol blue (0.01%) and β-Mercaptoethanol (1%), and incubated at 100 °C for 5 minutes. Equal amounts of lysates were separated by SDS-polyacrylamide gel electrophoresis, transferred onto polyvinylidene fluoride membrane (Merck Millipore), incubated with primary antibody (Sox2, AB5603, Millipore; c-Myc, #5605, Cell Signalling Technology; β-Actin, #4970, Cell Signalling Technology) and then with secondary antibody (Anti-rabbit IgG, HRP-linked Antibody, #7074, Cell Signalling Technology), and visualised using the ECL Plus Reagent Kit (GE Healthcare). Quantification of western blots was performed using ImageJ software (https://imagej.nih.gov/ij/).

### Statistical analysis

Statistical analysis was performed using MS Office Excel (Microsoft). Statistical significance was determined by paired two-tailed t-test at levels of P < 0.05 (*), P < 0.01 (**), and P < 0.001 (***).

## Additional Information

**How to cite this article**: Barta, T. *et al*. miRNAsong: a web-based tool for generation and testing of miRNA sponge constructs *in silico.* *Sci. Rep.* **6**, 36625; doi: 10.1038/srep36625 (2016).

**Publisher’s note**: Springer Nature remains neutral with regard to jurisdictional claims in published maps and institutional affiliations.

## Supplementary Material

Supplementary Information

## Figures and Tables

**Figure 1:**
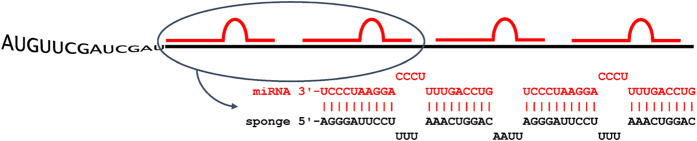
Schematic representation of miRNA sponge mechanism and design. Sponge transcript (black colour) contains high-affinity MBSs that sequester target miRNA (red colour).
