# Supplementary material for: miRNAsong: a web-based tool for generation and testing of miRNA sponge constructs in silico
Source: Sci Rep. 2016 Nov 18;6:36625. doi: 10.1038/srep36625 (PMC5114684; doi:10.1038/srep36625)
Supplement: Supplementary Information [file srep36625-s1.pdf]

Supplementary information file.

manuscript: miRNAsong: a web-based tool for generation and testing of miRNA sponge constructs *in silico*

Tomáš Bárta[1,2,*], Lucie Pešková[2], and Aleš Hampl[1,2]

[1]International Clinical Research Center, St. Anne's University Hospital Brno, Pekařská 53, 656 91 Brno, Czech Republic
[2]Department of Histology and Embryology, Faculty of Medicine, Masaryk University, Kamenice 3, 625 00 Brno, Czech Republic
*Corresponding author

Supplementary Data 1

Sanger sequencing of the vector with cloned miRNA sponge sequences (blue colour). Sequencing primer: CACCTGGCCCGATCTGGCC

tCnncTTtGCCTTTCTCTCcaCAGGTGTCcACTCCCAGGTCCaAGTTTGGTCTAGAGCTAGCCTGTGTACACTGGGGTCCC
AGGGATTCCTTTTAAACTGGACAATTAGGGATTCCTTTTAAACTGGACGGGTCCCAGGGATTCCTTTTAAACTGGACA
ATTAGGGATTCCTTTTAAACTGGACGGGTCCCCTGGCGGCCGCGCCCCTCTCCCTCCCCCCCCCCTAACGTTACTGGCC
GAAGCCGCTTGGAATAAGGCCGGTGTGCGTTTGTCTATATGTTATTTTCCACCATATTGCCGTCTTTTGGCAATGTGA
GGGCCCGGAAACCTGGCCCTGTCTTCTTGACGAGCATTCCTAGGGGTCTTTCCCCTCTCGCCAAAGGAATGCAAGGTC
TGTTGAATGTCGTGAAGGAAGCAGTTCCTCTGGAAGCTTCTTGAAGACAAACAACGTCTGTAGCGACCCTTTGCAGG
CAGCGGAACCCCCCACCTGGCGACAGGTGCCTCTGCGGCCAAAAGCCACGTGTATAAGATACACCTGCAAAGGCGG
CACAACCCCAGTGCCACGTTGTGAGTTGGATAGTTGTGGAAAGAGTCAAATGGCTCTCCTCAAGCGTATTCAACAAG
GGGCTGAAGGAtGCCCAgAAGGTACCCCATTGTATGGGATCTGATCTGGgGCCTCnGTGCacaTGCnTTtnCaTGtGTTT
AntCnAngntnnanAAAACgTCTangCCCcccgaa

Supplementary Figure S1

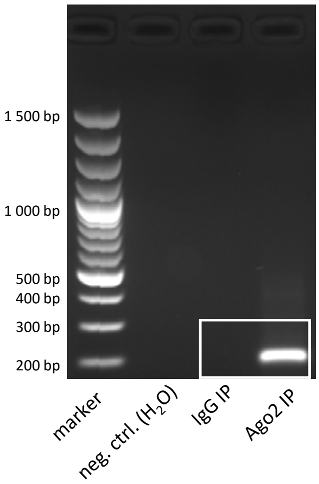

Supplementary Figure 1: Quantification of sponge transcript levels in Ago2-immunoprecipitated (Ago2 IP) and control IgG (IgG IP) fractions, as demonstrated by RT-qPCR. Full-scan of gel presented in the Figure 2B with molecular weight marker indicated. White rectangle shows cropped area used in the Figure 2B.

Supplementary Figure S2

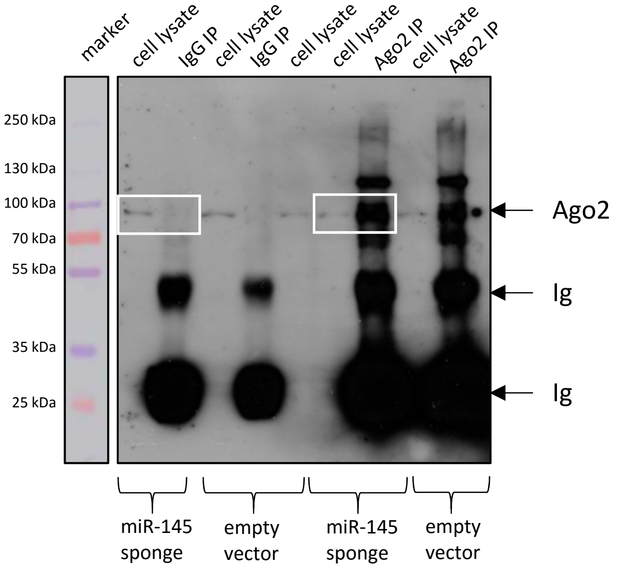

Supplementary Figure 2: Western blot analysis of Ago2 levels in Ago2 IP and control IgG IP fractions. Full-scan of blot presented in the Figure 2C with molecular weight marker indicated. Corresponding fragments of blot (white rectangles) used in the Figure 2C are indicated.

Supplementary Figure S3

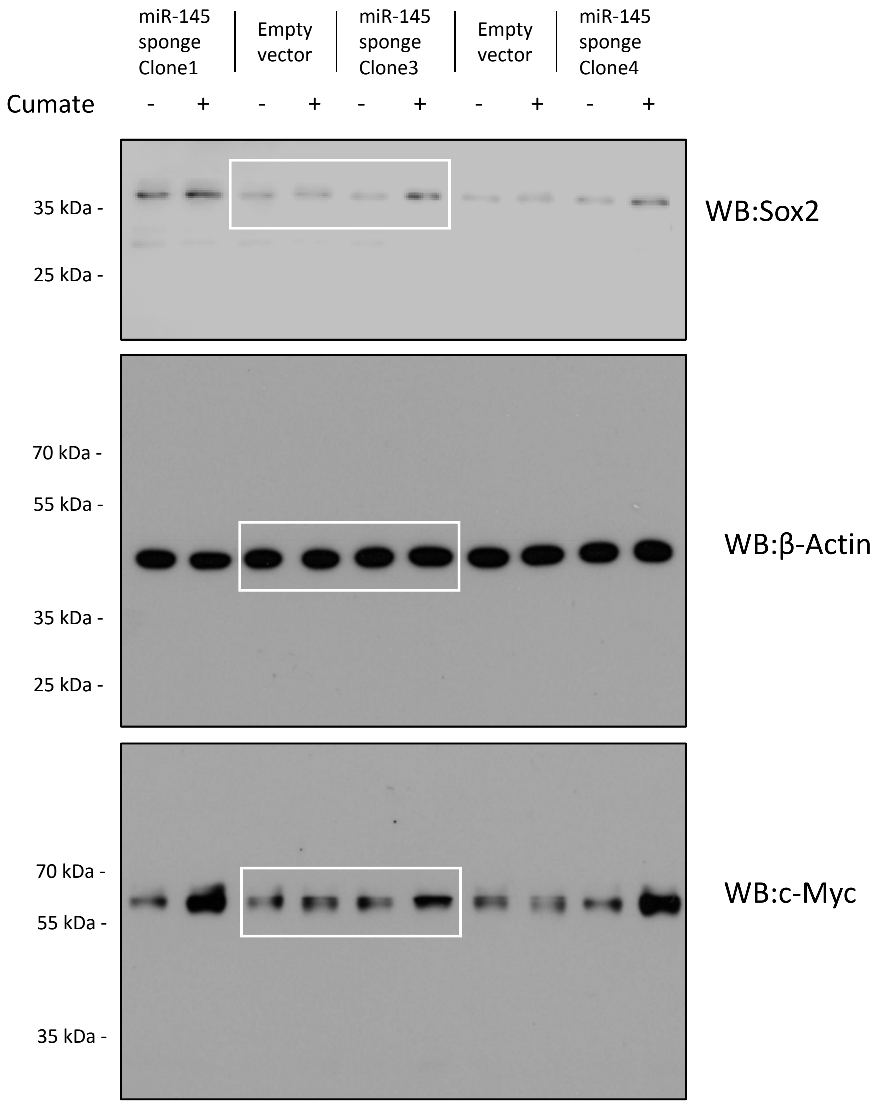

Supplementary Figure 3: Expression of c-Myc and Sox2 upon miR-145 sponge induction, as determined by western blot analysis. Full-scans of blots presented in the Figure 2D with molecular weight marker indicated. Corresponding fragments of blot (white rectangles) used in the Figure 2D are indicated.
